# Ability and Nonability Predictors of Real-World Skill Acquisition: The Case of Rubik’s Cube Solving

**DOI:** 10.3390/jintelligence11010018

**Published:** 2023-01-16

**Authors:** Elizabeth J. Meinz, David Z. Hambrick, James J. Leach, Prairie J. Boschulte

**Affiliations:** 1 Department of Psychology, Southern Illinois University Edwardsville, Edwardsville, IL 62026, USA; 2 Department of Psychology, Michigan State University, East Lansing, MI 48824, USA

**Keywords:** skill acquisition, expertise, cognitive ability, fluid intelligence, personality

## Abstract

Most research on skilled performance is correlational, with skill and predictors measured at a single point in time, making it difficult to draw conclusions about the acquisition of skill. By contrast, in this study, we trained novice participants (*N* = 79) to solve Rubik’s Cubes using a 7-step solution method. Participants also completed measures of fluid intelligence (Gf), working memory capacity (WMC), and nonability factors (grit, growth mindset, NFC, and the “big five”). Overall, higher Gf (but not WMC) was predictive of efficient and accurate Rubik’s cube skill. No nonability variables were associated with skill. Our results provide compelling evidence for the importance of intellectual talent (cognitive ability) in developing expertise in a complex task.

## 1. Background

Cognitive ability is a powerful predictor of real-world outcomes reflecting complex skill acquisition (Ackerman 1988; Kanfer and Ackerman 1989). As a case in point, meta-analyses consistently show that cognitive ability positively predicts job performance across a wide range of professions (Schmidt and Hunter 2004). Cognitive ability also predicts performance in specialized domains of expertise such as chess (Burgoyne et al. 2016), cryptic crossword puzzle solution (Friedlander and Fine 2020), and piano sight-reading (Meinz and Hambrick 2010). The influence of nonability factors such as personality and motivation have also been investigated, and, generally speaking, these factors are weaker or even nonsignificant predictors of performance after cognitive ability is controlled (e.g., Burgoyne et al. 2019).

Nevertheless, there are at least two major limitations of much research on the role of cognitive ability in complex skill acquisition. The first is that studies in this area often use a cross-sectional design: individuals representing different levels of skill are compared at a single point in time. In this type of study, conclusions can only be drawn about the relationship between skilled *performance* and cognitive ability, not skill *acquisition* and cognitive ability. A particular problem is selective attrition and the resulting restriction of range. For example, in job-performance studies, samples will include workers who have performed well, were motivated to continue, and had personality traits that were a good fit for the job. Similarly, in specialized domains, samples likely will not include those who quit because of poor performance, lack of motivation, or incompatible personality traits. Thus, it is possible that the roles of ability and nonability factors in performance may be underestimated due to the restriction of range.

The second limitation is that, when skill acquisition is studied using a longitudinal (training) approach, the research typically uses highly controlled laboratory tasks instead of complex real-world tasks (for a historical review, see Anderson et al. 2021). A notable exception is provided by Burgoyne et al. (2019). They trained a large sample of college students with no prior piano training to play two simple piano pieces using a standardized training protocol. A general intelligence factor accounted for more variance (21.4%) than any other predictor, whereas the effects of nonability factors were near zero. Thus, this study made use of a complex, real-world task and examined skill acquisition over time.

## 2. Present Study

Herein, we set out to investigate predictors of complex skill acquisition using the type of research approach employed by Burgoyne et al. (2019). The domain for our research was Rubik’s Cube solving. Invented in 1974 by the Hungarian architect and sculptor Erno Rubik, the classic puzzle was released internationally in the 1980s (www.ruwix.com; accessed on 1 November 2022) and became an iconic toy. Rubik’s Cube is an excellent task to use in studying complex skill acquisition in a few respects. It can be easily administered in the lab but is still relatively complex. Moreover, there are established approaches for learning the skill. For example, the popular “beginner method” can be used to successfully complete the cube by applying seven steps. As with many work tasks, the mastery of the first step allows one to move on to the next step, with notable interindividual variation in the rate at which each step is mastered. The solution of the cube via this method is difficult but not impossible in a limited amount of time, making it amenable to training in a laboratory setting. 

We tested for the effects of two cognitive-ability constructs on Rubik’s Cube skill. The first was *fluid intelligence* (Gf). Defined as the ability to solve novel problems (Cattell 1943), Gf is measured with tests of spatial visualization and abstract reasoning (e.g., Raven’s progressive matrices). We expected that Gf would positively predict Rubik’s Cube skill, given that reasoning processes must be used to evaluate the consequences of different moves. The second construct was working-memory capacity (WMC), which correlates highly with Gf but is a distinct cognitive ability (Kane et al. 2005; Chuderski 2013). WMC can be thought of as the ability to simultaneously store and process information, particularly in the presence of distraction (Hasher and Zacks 1988; Engle 2002), and is typically measured with “complex span” tasks (Conway et al. 2005), in which the participant’s goal is to perform a “processing” task (e.g., solving math equations) while also performing a “storage” task (e.g., remembering letters). Meinz and Hambrick (2010) found that WMC significantly predicted variability in piano sight-reading performance after controlling for measures of practice. We predicted that WMC would also positively predict Rubik’s Cube skill, given that, in learning how to solve the Rubik’s Cube, an individual must hold in mind the steps of an algorithm. 

We also tested whether nonability factors would add to the prediction of Rubik’s Cube skill, above and beyond Gf and WMC. We considered the following factors: (1) *growth mindset*: the belief that abilities are malleable rather than fixed (Dweck and Leggett 1988); (2) big five personality traits: neuroticism, extraversion, openness, agreeableness, and conscientiousness (McCrae and Costa 1987); (3) *need for cognition*: (Cacioppo and Petty 1982); and (4) *grit*: perseverance towards long-term goals (Duckworth et al. 2007). Cross-sectional studies of job performance have suggested that, of the big 5 personality constructs, conscientiousness is the best predictor across job types, but other personality constructs predict performance in select work domains (Kuncel et al. 2010). We similarly reasoned that some of these constructs might promote engagement with the task and therefore skill acquisition. Similarly, because need for cognition reflects one’s interest in intellectually challenging activities, it may be a precursor to task engagement and skill acquisition. Accordingly, Hambrick et al. (2007) found that NFC predicted knowledge of current events through a path that included interest and engagement with the news. We further reasoned that grit and a growth mindset might play a role in learning to solve the Rubik’s Cube in a time-limited, single-session study. Although the role of these nonability factors in skill acquisition has not been studied extensively, Burgoyne et al. (2019) found that neither growth mindset nor openness to experience made independent contributions to the acquisition of piano skill. Furthermore, although Kuncel et al. did find that personality variables predicted variance in job performance, their contributions were modest compared to the contribution of cognitive ability. Thus, we further predicted that effects of the nonability factors on Rubik’s Cube skill would be small and, even if statistically significant, smaller in magnitude than effects of Gf and WMC.

## 3. Method

### Participants

Participants (*N* = 79, 67.1% female, *M_age_* = 20.1) were recruited through undergraduate introductory psychology courses and received credit through their course as one of a few research-related assignment options. The pre-testing of a large pool of undergraduate students ensured that only those who reported little to no previous Rubik’s cube experience (either “1 = I have never played with a Rubik’s cube” or “2 = I have fiddled around with a Rubik’s cube, but probably not for more than a few hours ever” on a 7-point experience scale). Self-reported ACT test scores (i.e., a standardized test used for college admissions) ranged from 16.5 to 31 (with ACT conversions performed on three SAT scores), with a mean (*N* = 78, *M* = 21.7, *SD* = 3.3) only slightly higher than the national average at the time of the study (*M* = 20.8). 

## 4. Materials

**Need for cognition.** Need for cognition was measured with Cacioppo et al.’s (1984) 18-item scale. Each item is a statement such as *I prefer complex to simple problems* and *I only think as hard as I have to* (reverse scored). Using a 5-point scale, participants’ task is to rate how accurately each statement describes them. 

**Mini-International Personality Item Pool (Mini-IPIP).** The big five personality traits were measured with the Mini-IPIP (Donnellan et al. 2006). The scale includes 20 items, with four items for each trait (extraversion, agreeableness, conscientiousness, neuroticism, and openness). Using a 5-point scale, participants’ task is to rate how accurately each statement describes them compared to others of the same gender and approximate age. 

**Grit.** Grit was measured using Duckworth et al.’s (2007) scale. Each item is a statement such as *I finish whatever I begin* and *I often set a goal but later choose to pursue a different one* (reverse-scored). The measure was the average rating on the 1–5 scale (after re-coding negatively worded items).

**Growth mindset**. A 3-item, 6-point Likert scale measure (Chiu et al. 1997) was adapted to evaluate a participant’s self-reported mindset regarding their visuo-spatial ability (an ability that is relevant to learning perceptual–motor tasks such as the Rubik’s Cube; Fleishman and Rich 1963). For example, participants were asked to rate agreement with the statement: “your visual-spatial abilities are something about you that you cannot change very much.” Higher scores reflected a fixed mindset; lower scores reflected a growth mindset. Surprisingly, although participants’ ratings on the second and third items were highly correlated (*r* = .67), both of those items correlated only weakly with the first item (*r*s < .12).^1^ Therefore, we deemed the average rating of the second and third items to reflect *growth mindset*.

**Working memory capacity (WMC).** Working memory capacity (WMC) was assessed with two complex span tasks, administered via E-Prime (Oswald et al. 2015). In operation span, participants must solve a math equation and then remember a letter. After three to seven math–letter elements, they are required to recall the letters in the order in which they were presented. In symmetry span, participants must make a judgment about whether a pattern is symmetrical, then remember which cell of the matrix is filled. For each task, the score was the number of items on which both the memory and decision components were correct. 

**Fluid intelligence (Gf).** Fluid intelligence was measured with three tests. There were two tests of spatial visualization. The mental rotation test was a revised version of the Vandenberg and Kuse mental rotation test (Peters et al. 1995, Form A) consisting of 24 items. For each item, a target was presented on the left, and then four stimuli were presented on the right. Participants were to choose the two stimuli that were the same as the target, except that they were rotated on a vertical axis. Six min were given. The paper folding test (Ekstrom et al. 1976) consisted of 20 items on which participants inspected a target item (left column) illustrating a piece of paper that had been folded and then had holes punched in it. They then had to choose from five options on the right depicting what the hole pattern would look like when the paper was unfolded. Three min were given for each set of 10 items. For both tasks, participants’ scores were the % of items answered correctly. There was also a test of figural reasoning: Raven’s progressive matrices (Raven et al. 1998). In this test, participants are given a 3 × 3 matrix of figures with the lower-right figure missing. To choose the correct missing figure, participants must deduce the underlying rules that govern the progression of figures both vertically and horizontally in the matrix. Participants were given 10 min to solve 18 matrices. The score was proportionally correct.

**Rubik’s Cube training videos.** The Rubik’s Cube training course was based on what Rubik’s Cube enthusiasts call the *beginner method*. This method consists of 7 steps, each of which must be mastered before moving on to the next. Eight videos were produced by the same narrator for this study, including an introductory teaching session and one training video for each of the seven steps. This ensured equivalent training for all participants. 

The introductory video (11 min, 40 s) provided a basic overview of the Rubik’s Cube, describing important terminology and concepts needed for success in the training videos which would follow. In the Step 1 training video (10 min, 52 s), participants were instructed in how to make a white cross (or plus sign) on the white side of the cube. The Step 2 video (8 min, 17 s) described how to finish solving the white side of the Rubik’s Cube. As with each of the following training videos, Step 2 included training on how to use a specific algorithm (a predetermined series of moves to be performed in sequence) needed to solve the step. The Step 3 (13 min, 24 s) video trained participants to solve the middle layer of the cube, aligning the middle cube positions with their corresponding colors. The Step 4 video (7 min, 9 s) trained participants to complete a yellow cross (which is the opposing side to the completed white side of the cube). The Step 5 video (5 min, 44 s) taught participants how to align the sides/edges of the yellow cross so that they were the correct color. The Step 6 video (6 min, 14 s) went over how to correctly align the yellow corner pieces to their proper places (albeit not necessarily rotated correctly). Finally, the Step 7 (3 min, 36 s) video shows how to correctly align the four edge pieces of the yellow side, which results in a completely solved Rubik’s Cube. Table 1 provides an image of the cube after solving each step and the pieces possible for each step of the cube.

**Rubik’s Cube training video quizzes.** Following each of the eight videos, participants were given a 7-item, multiple-choice quiz that ensured the comprehension of the training content covered in the corresponding video. 

## 5. Procedure

Participants were tested individually in a single session lasting no longer than 6 h. The session length depended on performance, and participants were encouraged to take breaks between tasks. Participants signed an informed consent form and then completed the demographic questionnaire, followed by the nonability scales, the Gf tests, and the WMC tasks. All instruments were administered via computer in single-participant sessions. (The study was approved by the IRB at the first author’s institution.) 

Participants then began the training course with the introductory video. For each of the eight videos, participants were given a Rubik’s Cube, which allowed them to follow along with the steps in the video while watching. Immediately following each video, participants were given a quiz over the video content. Participants who did not pass (6/7 correct) the quiz on the first attempt were given the opportunity to re-watch the video and attempt the quiz again. Those who were unable to pass the quiz after three attempts were shown the correct answers and allowed to progress. Following the quiz for each of the Steps 1–7 videos, participants were given an unrestricted opportunity to practice while completing that step on their cubes. Practice time included the ability to re-watch the video of the current step (with pause, rewind, and fast-forward controls) while rehearsing step completion using the Rubik’s Cube. Participants were encouraged to practice as long as they wished and were asked to notify the experimenter when ready to begin assessment.

Assessment consisted of repeated tests (six per step) in which participants were asked to solve a Rubik’s Cube for the step on which they were just trained. Scrambled cubes vary in difficulty; it may be easier to solve one scrambled cube than another (scrambled differently). Therefore, the six cubes to be solved for each step were precisely arranged in the same scrambles for all participants. In other words, for each step, we pre-determined the arrangements of the six scrambled cubes, and each participant received cubes that were scrambled the exact same way. These scrambles were created using a scramble generator tool (http://rubikscube.info/pravidla/ (accessed on 1 August 2020) designed for this purpose. Participants’ solution time (up to a maximum time allotment for each step) and the number of pieces correctly placed were recorded for each of the six cubes. After testing on each cube, they were given the opportunity to practice again (under the same conditions as before for an unrestricted amount of time) before testing with the next scrambled cube.

Most participants did not complete all steps of the cube solution. This was either because they reached the 6 hr time limit before all steps were learned (*n* = 35), because they were respectfully dismissed because they did not solve at least one of the six cubes for that step (*n* = 39), or, in a few cases, because they chose to withdraw at some point during the cube-solution portion of the study (*n* = 4). 

## 6. Measures of Rubik’s Cube Performance

We computed multiple measures of Rubik’s Cube performance. The first measure, to capture overall performance, was *last step solved*: the number of steps of the Rubik’s Cube out of seven a participant successfully solved at least one time. The second measure, to capture the amount of time it took to achieve this level of performance, was *efficiency*: the sum of total time spent working to solve all cubes attempted (up to six cubes in each of seven steps), in seconds, divided by the total number of pieces attempted that were solved correctly. Thus, a lower efficiency score represents less time needed (s) for the participant to place a single piece correctly (therefore a lower efficiency score represents a *better* level of performance). The final measure, to capture the accuracy of all attempted solutions, was *accuracy*: the total number of pieces solved correctly divided by the total number of pieces attempted (also see Footnote 2). This is a measure of accuracy that is not based on the possible number of points but rather the number of points (pieces correct) the participant completed within the confines of the 6 h session. 

## 7. Results

To prepare the data for analysis, we screened all variables for outliers (>3 SD from the mean) and skew, but there were none. Across the predictor variable composites and three measures of performance, there were missing values for three participants on two of the performance variables and for five participants on either the operation or symmetry span. Because Little’s MCAR suggested that there was no consistent pattern to these missing values, ***X***^2^ (78, *N* = 80) = 92.80, *p* = .12, the expectation maximization (EM) procedure in SPSS was used to replace the performance variable values using data from all three performance variables across all seven steps. For participants with missing data on one of the WMC tasks, their data from the remaining task was used as their WMC value. 

Descriptive statistics and reliability for all ability and nonability variables can be found in Table 2. The Gf measures correlated positively and moderately with each other (avg. *r* = .49), as did the WMC measures (*r* = .37). For subsequent analyses, we created Gf and WMC composite variables by averaging the z scores for the variables corresponding to each factor.

**Figure 1:**
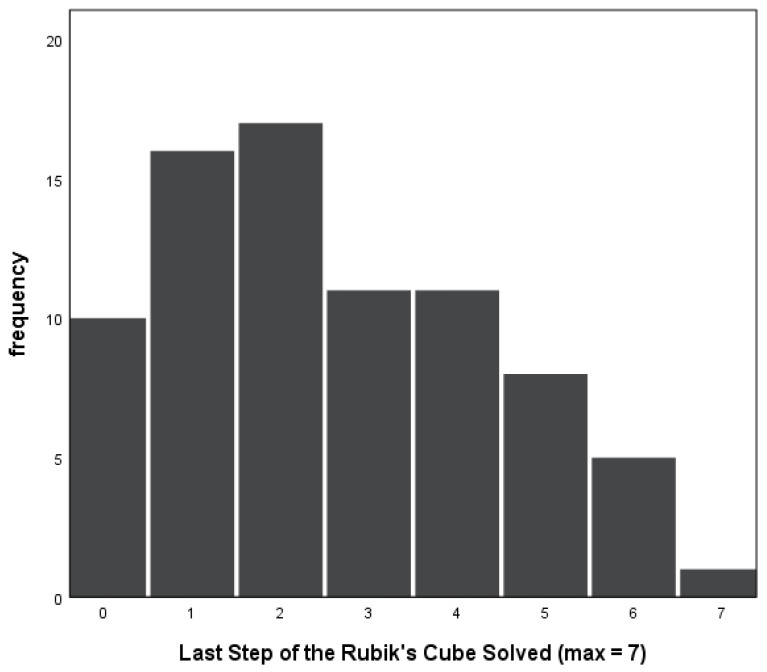
Histogram illustrating number of participants reaching each step.

As illustrated in Figure 1, the number of steps correctly completed ranged from zero to seven (*M* = 2.58, *SD* = 1.83). Participants placed between 24% and 100% *of the pieces that they attempted* correctly, with an average of 77% of attempted pieces placed correctly. This suggests that participants typically did not attempt to solve the cube until they were confident that they would perform well. 

## 8. Correlations

Correlations of the Rubik’s performance variables with the ability and nonability variables can be found in Table 3. (For all performance variables except efficiency, higher scores reflect better performance; because low efficiency scores reflect good performance, the correlations of this variable with the others were negative.) The Gf correlations were all statistically significant, whereas the WMC correlations only approached significance (*p*s = .014–.039). In striking contrast, the nonability correlations were all near zero. In sum, higher levels of cognitive ability, and especially Gf, correlated with greater success in learning how to solve the Rubik’s Cube.

### Regression Analyses

With a separate analysis for each Rubik’s Cube performance variable, we performed a series of hierarchical regression analyses with Gf entered in Step 1, WMC entered in Step 2, and the nonability variables entered in Step 3. Results are presented in Table 4. As expected, Gf accounted for a significant proportion of the variance in each performance variable: efficiency (30.9%), last step solved (34.7%), and accuracy (29.8%). However, contrary to our prediction, the effect of WMC was non-significant for each performance variable. Finally, nonability variables’ (entered as a group) effects were near zero. In fact, the strongest effect of a nonability variable on efficiency (Mindset β = .21, *p* = .08) was in the direction opposite to that predicted (i.e., lower efficiency for participants with a higher growth mindset). In sum, high levels of Gf were associated with better Rubik’s Cube learning. 

## 9. Discussion

What predicts the acquisition of a complex, real-world skill in novices when training is consistent and standardized? Our results suggest that cognitive ability—and more particularly abstract reasoning and spatial abilities (Gf)—plays a strong role (*r*s = .56–.59). Although previous authors have also argued for a role of cognitive abilities in skilled performance, most studies utilizing real-world tasks were conducted utilizing cross-sectional approaches that potentially confounded cognitive ability, practice, and skilled performance and suffered from selective attrition. Therefore, it is not surprising that the size of the effect herein is larger than those reported in meta-analyses of cross-sectional studies of chess skill (*r* = .24; Burgoyne et al. 2016) or musical achievement (*r* = .25; Ruthsatz et al. 2008); however, the effect size is comparable to those in a meta-analysis of athletic performance (Hedges *g* = .58; Kalén et al. 2021). 

WMC did not predict the acquisition of Rubik’s Cube skill. Previously, WMC did predict performance in music sight-reading among pianists of a wide range of skill (Meinz and Hambrick 2010) and in novices’ ability to learn to play the piano (Burgoyne et al. 2019). In the latter study, WMC was part of an ability composite. Instead, we chose to consider its influence separately. The data supported this decision, as although the WMC and Gf composites correlated positively (*r* = .27), this correlation was weaker than the correlation between the two working memory measures (*r* = .37) and the correlations among the spatial ability and reasoning measures (*rs* = .43–.57). Meinz and Hambrick speculated that WMC might allow pianists to plan ahead for future finger movements when sight-reading (i.e., playing a never-before-seen piece of music) by looking ahead in the music. Similarly, we reasoned that Rubik’s Cube solution similarly might benefit from thinking ahead to the next algorithm. It is also possible that WMC might be helpful when one is monitoring how far along they are in the solution algorithm (as some are lengthy). Nonetheless, WMC did not predict performance here. 

Here, neither personality (big 5, need for cognition) nor grit or mindset predicted skill acquisition, individually or as a whole. These findings are largely consistent with previous research (e.g., Ackerman et al. 1995; Burgoyne et al. 2019), suggesting these types of nonability factors might not predict skill acquisition after cognitive ability is considered.

**Figure 2:**
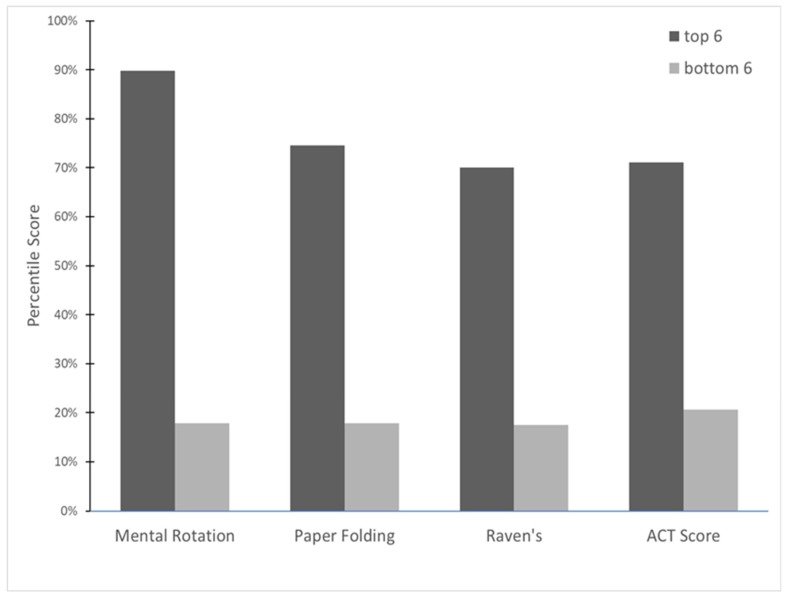
Relationship between Gf and Rubik’s Cube efficiency. *Note.* Average percentile score on Gf tests and ACT for top (*n* = 6) vs. bottom (*n* = 6) participants on Rubik’s Cube solution efficiency. (For ACT, the top group includes only 5 participants because one participant did not report ACT score).

In sum, we presented data from a large sample of novice participants who ranged widely in cognitive ability and nonability variables (see Table 2). These participants were trained, using a consistent protocol, to solve the Rubik’s cube. Nonability variables (personality, grit, mindset) did not predict skill acquisition. Instead, our results emphasize the role of basic cognitive ability in complex skill acquisition, with between 30% and 35% of the variance in Rubik’s Cube performance variables accounted for by a composite derived from measures of reasoning and spatial ability. In a particularly compelling visualization, Figure 2 illustrates the sample-based percentile scores for ACT, both measures of spatial ability (paper folding and mental rotation), and reasoning (Raven’s) for the top six and the bottom six Rubik’s cube performers. Because our participants were novices at the beginning of the session, ability effects did not reflect selective attrition in the domain, and training was the same for all participants. We therefore conclude that the acquisition of skill in some domains may be largely driven by cognitive ability, and not by practice, personality, a particular type of mindset, or the tendency to persist in the face of challenge. 

One strength of this study is also its caveat: the use of a single, 6 h session. Because participants had agreed to participate in a 6 h training session, the role of personality characteristics in skill acquisition might have been reduced due to a selection bias. Additionally, because participants were released from the study if they did not complete the cubes within the time limit, the role of some personality characteristics (e.g., grit) might have been attenuated. Future researchers should aim to more fully explore the ways in which the skill acquisition context could influence personality-skill relations. Second, without additional sessions, it was not possible to determine whether the role of Gf in skill acquisition declines as participants reach high levels of skill, as suggested by Ackerman (1988). There were simply too few participants who reached the last steps to conduct Step X Gf interaction analyses on the performance variables. We assert that Rubik’s Cube solution is a domain in which skill acquisition can be studied with reliable measures yielding a wide range of performance, easy access to novice participants, and skills that may be attainable in a reasonable timeframe. Thus, we encourage future researchers to further investigate this domain when considering the intersecting roles of accumulating practice, cognitive ability, and personality. 

## Figures and Tables

**Table 1:** Summary of Each Step and Performance.

Step	Picture(s)	Max # of Pcs to Be Placed Correctly to Solve This Step^2^	% of Total N Who Solved	Fastest Solution Time (s)	Max Allotted Time (s) per Cube to Complete through This Step	Accuracy
1	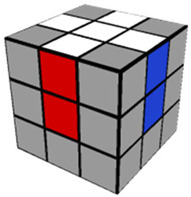	4	87.3	10.0	300.0	81.8
2	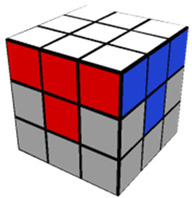	8	67.1	20.0	480.0	84.7
3	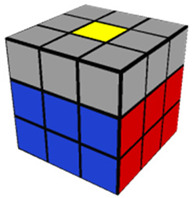	12	46.8	120.0	600.0	86.1
4	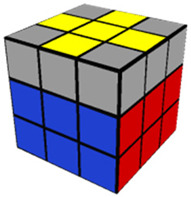	16	31.6	117.1	600.0	93.9
5	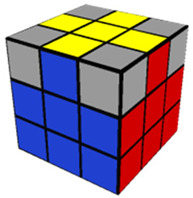	20	17.7	140.7	600.0	95.7
6	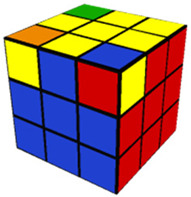	24	7.6	161.0	600.0	97.1
7	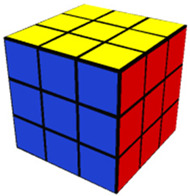	28	1.3	293.1	600.0	50.0 *

*Note.* 10 participants (12.7%) failed to solve Step 1; % who solved = % of participants who solved the cube within 6 attempts; Accuracy = the number of pieces solved divided by the number of pieces attempted. Images from https://solvethecube.com/ (accessed on 1 November 2022). (* This mean is from a single participant who solved 0 pieces on the first testing attempt and then solved the entire cube perfectly in the second attempt because they made a mistake at the end of the timed testing period and attempted to start over from the beginning).

**Table 2:** Descriptive statistics for personality and ability.

	M	SD	min	max	Possible	Reliability
**Ability**						
**Gf z-composite**	**.00**	**.81**	**−1.48**	**2.04**	**-**	**-**
Mental rotation (Gf)	.38	.20	.04	.88	0–1	.82
Paper folding (Gf)	.50	.17	.10	.90	0–1	.74
Ravens (Gf)	.43	.15	.06	.83	0–1	.62
**WMC z-composite**	**.78**	**1.11**	**−2.32**	**1.73**	**-**	**-**
Operation span (WMC)	22.14	6.20	3	30	0–36	-
Symmetry span (WMC)	14.04	4.55	5	24	0–36	-
**Personality**						
Need for cognition	3.27	0.56	1.78	4.28	1–5	.85
Grit	3.29	0.50	1.78	4.28	1–5	.73
Mindset	3.58	1.01	1.00	5.50	1–6	.80
Extroversion	3.03	0.97	1.00	5.00	1–5	.80
Agreeableness	3.97	0.61	2.50	5.00	1–5	.49
Conscientiousness	3.50	0.83	1.50	5.00	1–5	.72
Neuroticism	2.76	0.78	1.00	4.50	1–5	.55
Openness	3.81	0.62	2.25	5.00	1–5	.59
**Performance**						
Last step solved	2.58	1.83	0	7	0–7	-
Efficiency (s/piece)	72.39	63.86	14.62	291.67	0–22,680	-
Accuracy	.77	.23	.24	1	0–1	-

*Note.* If a participant used the maximum allotted time (5 min) for each of the 6 cubes in Step 1, the possible range for Step 1 solution time would be 0–1800 s. Therefore, summing the max allotted times listed in Table 1 for the 6 cubes in each step, the maximum solution time across all steps would have been 22,680 s or 378 min. The scores for the mental rotation, paper folding, and Raven’s reflect proportion correct; Working memory scores reflect the number of items correct; scores on the personality measures are the average of all Likert-scale items (higher numbers reflect higher levels of the trait). Reliability = Cronbach’s alpha. Due to a data recording error, we could not compute Cronbach’s alpha for the WMC tasks. However, the WMC composite apparently had good reliability because the ability and nonability predictors collectively accounted for 37% of the variance in WMC performance (multiple R = .61).

**Table 3:** Correlations of predictor variables with performance variables.

	Rubik’s Performance Variables		
**Rubik’s Performance Variables**	1.	2.	3.
1. Accuracy	**--**		
2. Efficiency	**−.634**	**--**	
3. Last Step Solved Correctly	**.960**	**−.734**	**--**
**Predictor Variables**			
4. Gf	**.546**	**−.556**	**.589**
5. WMC	.268	−.233	.236
6. NFC	.133	.082	.136
7. Grit	.082	.054	.083
8. Mindset (higher = fixed)	−.122	.218	−.189
9. Neuroticism	−.058	.147	−.121
10. Extroversion	−.109	.148	−.069
11. Openness	.001	.148	−.021
12. Agreeableness	−.045	.094	−.029
13. Conscientiousness	−.032	.138	−.081

*Note.* Scores for the Gf and WMC measures reflect proportion correct; scores for the nonability measures were average Likert ratings (higher numbers reflect higher levels of the trait). Bold values indicate *p* < .01.

**Table 4:** Results of hierarchical regression analysis.

	*Performance Variable*	*ΔR^2^*	*ΔF*	*df*
***Step 1: Gf***				
	*Efficiency*	*.309*	33.13 **	*1,74*
	*Last Step Solved*	*.347*	40.92 **	*1,77*
	*Accuracy*	*.298*	31.43 **	*1,74*
***Step 2: WMC***				
	*Efficiency*	*.008*	*0.84*	*1,73*
	*Last Step Solved*	*.006*	*0.71*	*1,76*
	*Accuracy*	*.061*	*1.74*	*1,73*
***Step 3: Nonability factors***				
	*Efficiency*	*.070*	*0.61*	*8,65*
	*Last Step Solved*	*.039*	*0.55*	*8,68*
	*Accuracy*	*.039*	*0.49*	*8,65*

*Note.* Gf, fluid intelligence composite. WMC, working memory capacity composite. Nonability factors, need for cognition, grit, growth mindset, neuroticism, extraversion, openness, agreeableness, and conscientiousness. ** *p* < .001.

## Data Availability

Data are available by request from first author.

## Notes

1 We acknowledge that this is an unusual finding. As other researchers have done when using these items, we inserted words into the statements to better reflect the content of our study. The items, in order, were as follows: “You have a certain amount of ability to solve visual-spatial problems (for example, following picture instructions on how to put together a piece of furniture, navigating in an unfamiliar city, imagining how options would look from different angles), and you can’t really do much to change it”, “Your visual-spatial abilities are something about you that you can’t change very much”, “Being a “visual-spatial person” or not is something that you really can’t change. Some people are good at it and other people aren’t.” It is not clear to us why the reliability of the 3 items was poor.

2 Note that an adjustment of the recorded number of pieces attempted and solved correctly for Steps 6 and 7 was necessary for calculation purposes. These values varied linearly for Steps 1–5. For example, the possible number of pieces correct on Step 1 was 4 (the pieces that make a “White Plus”, disregarding the fixed middle piece on the cube). For Steps 2–5, the possible number of pieces correct were 8, 12, 16, and 20, respectively. However, because the possible number of correct pieces for the sixth and seventh steps was also 20 (as these steps involve a realignment of the pieces rather than a position change), we transformed these values by a factor to make the possible numbers of correct for Steps 6 and 7 twenty-four and twenty-eight, respectively. This created a scoring system in which accuracy on each step reflects a 4-point gain from the previous step and therefore a wide range of possible scores on accuracy and greater variability in participants’ scores.
